# Translating whole‐genome doubling into precision medicine in cancer

**DOI:** 10.1002/1878-0261.70302

**Published:** 2026-07-12

**Authors:** Sejung Lee, Junghyeok Lim, Jinhyuk Bhin

**Affiliations:** ^1^ Department of Biomedical Systems Informatics Gangnam Severance Hospital, Yonsei University College of Medicine Seoul Republic of Korea; ^2^ Graduate School of Medical Science, Brain Korea 21 Project Yonsei University College of Medicine Seoul Republic of Korea

**Keywords:** cGAS‐STING, chromosomal instability, polyploidy, precision oncology, tumor evolution, whole‐genome doubling

## Abstract

Whole‐genome doubling (WGD) occurs in approximately 30–40% of human cancers, representing a transformative evolutionary event that fundamentally reshapes tumor biology. WGD triggers chromosomal instability (CIN) and somatic copy‐number alterations (SCNAs), which accelerate tumor evolution and underlie aggressive clinical behavior. Paradoxically, the same cellular stresses that drive tumor progression also force WGD‐positive cells into a heightened dependence on specific survival mechanisms, meaning that poor prognosis and therapeutic vulnerability arise from the same underlying phenomenon. Despite the widespread occurrence and clinical impact of WGD, translating its biology into actionable therapeutic strategies remains limited due to the lack of prospective evidence and integrated biomarker frameworks. This review provides a comprehensive overview of WGD biology, from mechanisms to clinical applications, examining how WGD arises and drives tumor evolution, its prevalence and prognostic impact across cancer types, and the emerging therapeutic vulnerabilities that WGD‐positive tumors expose.

## Graphical abstract

Whole‐genome doubling creates a WGD‐positive tumor state characterized by persistent chromosomal instability, karyotypic diversification, and cellular stress. These same biological pressures drive aggressive tumor evolution while exposing therapeutic vulnerabilities, providing a rationale for WGD‐informed precision medicine.

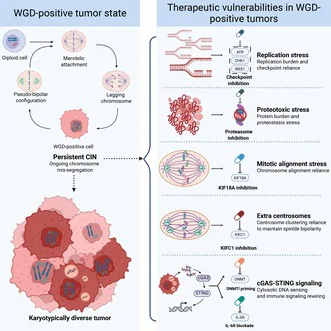

**Abbreviations**
APC/C: anaphase‐promoting complex/cyclosome
ATR: ataxia–telangiectasia and Rad3‐related (protein kinase)
CCNE1: cyclin E1
CCNE2: cyclin E2
CDK1: cyclin‐dependent kinase 1
CDKN2A: cyclin‐dependent kinase inhibitor 2A
cGAS: cyclic GMP–AMP synthase
CHK1: checkpoint kinase 1
CIN: chromosomal instability
DNMTi: DNA methyltransferase inhibitor
ESCRT: endosomal sorting complex required for transport
HGSOC: high‐grade serous ovarian cancer
HMF: Hartwig Medical Foundation
IL‐6: interleukin‐6
KIF18A: kinesin family member 18A
KIFC1: kinesin family member C1
LOH: loss of heterozygosity
LUAD: lung adenocarcinoma
LUSC: lung squamous cell carcinoma
MHC‐II: major histocompatibility complex class II
MSK‐IMPACT: Memorial Sloan Kettering‐Integrated Mutation Profiling of Actionable Cancer Targets
NF‐κB: nuclear factor kappa‐light‐chain‐enhancer of activated B cells
NMIBC: nonmuscle‐invasive bladder cancer
NSCLC: nonsmall‐cell lung cancer
PCAWG: Pan‐Cancer Analysis of Whole Genomes
SCNA: somatic copy‐number alterations
STAT3: signal transducer and activator of transcription 3
STING: stimulator of interferon genes
TCGA: The Cancer Genome Atlas
WEE1: WEE1 G2 checkpoint kinase
WGD: whole‐genome doubling

## 1 Introduction

Whole‐genome doubling (WGD) is a transformative event wherein a cell's entire chromosomal content is doubled, resulting in polyploidy [1, 2, 3]. Physiologically, polyploidization is a regulated developmental process restricted to specific cell lineages such as hepatocytes, cardiomyocytes, and megakaryocytes, serving adaptive functions that include increased metabolic capacity and terminal differentiation [4, 5, 6]. In normal development, polyploid cells are prevented from re‐entering the cell cycle by epigenetic mechanisms. In hepatocytes, atypical E2F repressors silence mitotic and cytokinesis genes [7, 8]. In cardiomyocytes, H3K9 trimethylation epigenetically silences mitotic and cell‐cycle genes [9, 10]. Because this mitotic program is kept off, polyploid cells do not return to a proliferative state and therefore do not progress to a transformative phenotype. Unlike the stable and balanced polyploidy observed in normal tissues, cancer‐associated WGD typically precipitates extensive chromosomal instability (CIN) and aneuploidy [1, 11] and consequently acts as a key driver of tumor evolution [1, 12, 13, 14].

Mechanistically, WGD in cancer often arises from cell division failure, generating polyploid cells that must overcome significant hurdles to survive, including supernumerary centrosomes and heightened replication stress [1, 3, 11, 15]. A critical trade‐off accompanies this process. While WGD can provide a buffering effect against deleterious copy‐number alterations, it simultaneously imposes severe burdens on cellular homeostasis [16, 17]. The net impact of WGD on tumor fitness is therefore not uniform but highly context‐dependent, varying substantially according to the genetic background and epigenetically regulated programs, particularly those shaping immune sensing [14, 18, 19].

Reflecting this biological complexity, WGD‐positive tumors exhibit distinct but heterogeneous clinical phenotypes across cancer types [12, 20]. Although WGD is generally associated with poor prognosis, its clinical implications are modulated by diverse factors, including the tissue of origin, mutational landscape, and the tumor microenvironment [12, 14]. While our mechanistic understanding of WGD has advanced considerably, translating this knowledge into actionable clinical strategies remains a critical unmet need [2, 12, 14].

This review provides a comprehensive overview of WGD biology from mechanisms to clinical applications, examining how WGD arises and drives tumor evolution, its prevalence and prognostic impact across cancer types, and the emerging therapeutic vulnerabilities exposed by WGD‐positive tumors.

## 2 WGD as an evolutionary engine in cancer

### 2.1 Chromosomal instability and karyotypic diversification

WGD constitutes a macro‐evolutionary leap in the trajectory of tumor progression. Rather than representing a mere accumulation of local alterations, WGD propels a lineage into a distinct genomic state that fundamentally alters the fitness landscape [12]. By doubling the chromosomal content, WGD permits the exploration of karyotypic configurations that are inaccessible to diploid cells, thereby increasing tumor genome complexity and ultimately driving tumor evolution (Fig. 1A) [1, 17, 21]. Phylogenomic analyses consistently place WGD as an early truncal event followed by the progressive accumulation of somatic copy‐number alterations (SCNAs) and large‐scale loss of heterozygosity (LOH) events, positioning it as a critical inflection point that initiates a trajectory of ongoing CIN and accelerated karyotypic evolution [1, 2, 12]. The mechanistic basis of this instability lies in the inherent disruption of mitotic fidelity by the polyploid state. The acquisition of supernumerary centrosomes following WGD promotes the formation of transient multipolar spindles, which, even when resolved into pseudo‐bipolar configurations, frequently produce merotelic kinetochore attachments and lagging chromosomes [11, 22]. Critically, because centrosome number is not corrected by pseudo‐bipolar resolution, this error‐prone configuration persists across successive divisions, establishing a self‐perpetuating cycle of chromosome mis‐segregation rather than a transient consequence of the initial genome doubling [1, 23]. The resulting karyotypic diversification, including recurrent patterns of arm‐level copy‐number changes observed across cancer types [24], generates extensive intra‐tumoral heterogeneity that provides the substrate for clonal selection [1, 21, 25].

**Fig. 1:**
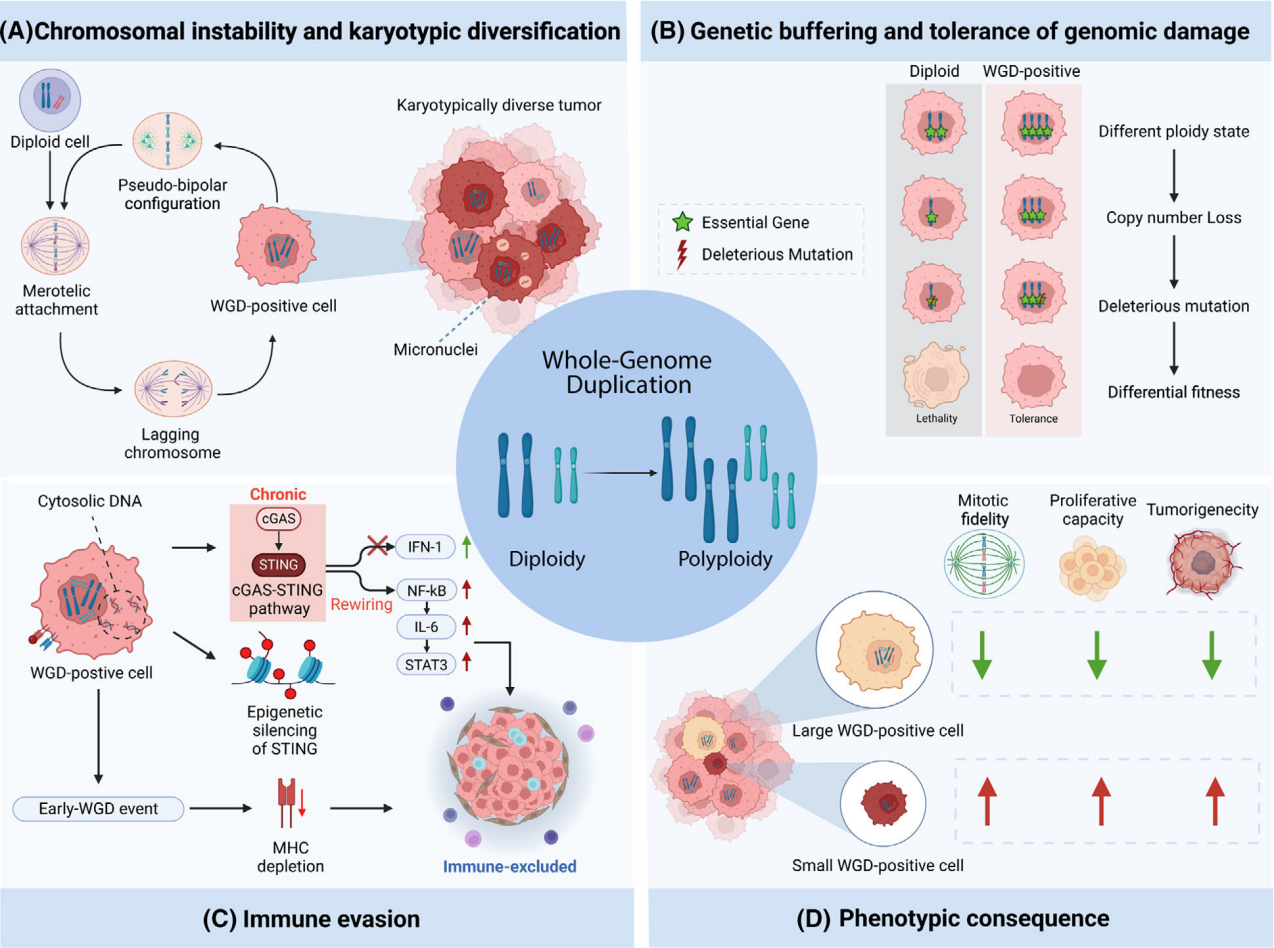
WGD as an evolutionary engine in cancer. WGD reshapes tumor evolution through several interconnected consequences. (A) Supernumerary centrosomes after WGD generate persistent chromosome mis‐segregation and lagging chromosomes, producing micronuclei and karyotypic diversification. This self‐perpetuating cycle of CIN generates diverse karyotypes that provide the substrate for clonal selection. (B) Gene‐dosage redundancy from WGD buffers essential gene loss (green star) and deleterious mutations (red bolt) that would be lethal in diploid cells. This expanded tolerance enables clones to sustain otherwise lethal chromosome losses. (C) WGD‐driven CIN generates cytosolic DNA that engages the cGAS–STING pathway, but chronic activation attenuates type I IFN signaling while NF‐κB/IL‐6/STAT3 immunosuppressive programs remain active. STING can be epigenetically silenced, and early WGD is associated with MHC‐II depletion. These mechanisms promote an immune‐excluded tumor microenvironment. (D) Small WGD‐positive cells show improved mitotic fidelity, proliferative capacity, and tumorigenicity (green arrows) compared to large size‐scaled polyploid cells (red arrows). This size‐dependent fitness variation contributes to phenotypic selection within WGD‐derived lineages. cGAS, cyclic GMP–AMP synthase; CIN, chromosomal instability; IFN, interferon; IL‐6, interleukin‐6; MHC, major histocompatibility complex; NF‐κB, nuclear factor κB; STAT3, signal transducer and activator of transcription 3; STING, stimulator of interferon genes; WGD, whole‐genome doubling.

### 2.2 Genetic buffering and tolerance of genomic damage

WGD‐driven CIN continuously generates karyotypic diversity, but the evolutionary impact of this diversity depends on whether cells can survive the resulting genomic perturbations (Fig. 1B) [1]. In a diploid context, chromosome loss is frequently lethal because it can eliminate the remaining functional copy of essential genes through LOH [17] and disrupt the stoichiometric balance of multi‐protein complexes, generating proteotoxic stress [26, 27]. The gene‐dosage redundancy inherent in the duplicated genome mitigates both of these vulnerabilities by maintaining additional copies of critical loci, thereby expanding the range of tolerable karyotypic states [1, 17]. This tolerance also extends to deleterious point mutations, as extra gene copies can mask the functional impact of mutations in essential genes.

Beyond immediate damage tolerance, this buffering can facilitate tumor evolution by allowing clones to sustain large‐scale LOH events that would be lethal in a diploid background, thereby enabling the complete inactivation of tumor‐suppressor genes [1]. Consistent with this, WGD‐positive tumors accumulate significantly more LOH than their diploid counterparts across cancer types, and the extent of post‐WGD copy‐number loss correlates with the degree of initial ploidy gain [17]. Together, these observations suggest that genetic buffering is not merely a passive survival mechanism but an active enabler of the genomic diversification that underlies tumor adaptation.

### 2.3 Immune evasion

The evolutionary advantages conferred by WGD extend beyond cell‐intrinsic genomic mechanisms to encompass the remodeling of the tumor–immune interface (Fig. 1C) [14, 18, 19]. The CIN driven by WGD generates cytosolic DNA through chromosome mis‐segregation and micronuclei rupture, which would be expected to activate the cGAS–STING innate immune sensing pathway and induce interferon‐linked programs [13, 28, 29]. However, CIN‐high and aneuploid states are frequently associated with immune evasion features and reduced immunotherapy response, suggesting that immune selection pressure acting on WGD‐positive clones can favor the outgrowth of cells that have uncoupled chromosomal instability from effective immune activation [14, 18].

This immune evasion can arise through distinct mechanisms. Persistent CIN associated with WGD drives chronic cGAS–STING activation, which paradoxically becomes rewired away from productive type I interferon signaling. Instead, it engages ER‐stress‐associated and NF‐κB/IL‐6‐driven inflammatory programs, promoting an immune‐suppressive tumor microenvironment permissive for progression [30, 31]. In several cancers, tumor‐intrinsic cGAS–STING signaling is attenuated through promoter methylation and repressive chromatin states, though whether such silencing is specifically associated with WGD‐positive tumors remains to be established [32, 33, 34, 35]. In addition, the timing of WGD during tumor evolution can affect immune evasion phenotypes. In serous ovarian cancer, tumors with earlier WGD show greater depletion of MHC class II expression than those with late WGD, consistent with prolonged post‐WGD evolution allowing more extensive immune‐evasive remodeling [19]. Together, these observations suggest that WGD‐positive tumors can co‐evolve with immune‐evasive adaptations, enabling the expansion of clones with extensive genomic alterations that would otherwise be subject to immune‐mediated elimination [14, 18, 19, 33].

### 2.4 Cell size variation and fitness heterogeneity

WGD can also influence tumor evolution through phenotypic variation, such as differences in cell size scaling, that impact fitness (Fig. 1D). Genome doubling typically increases cell size in proportion to DNA content, but this scaling is not universal [36]. A subset of polyploid cells can remain relatively small after genome doubling, and these cells can display improved mitotic fidelity, enhanced cellular fitness, and increased tumor‐forming capacity compared with size‐scaled polyploids [37]. Although the mechanistic basis of this size‐fitness relationship remains to be determined, this pattern suggests that WGD‐positive cells can harbor selectable variation in how cell size scales with ploidy, contributing to fitness heterogeneity within WGD‐derived lineages.

## 3 Cellular and molecular mechanisms of WGD

### 3.1 Cell‐cycle routes to polyploidization

#### 3.1.1 Mitotic slippage after prolonged mitotic arrest

Mitotic slippage is defined as a time‐dependent escape from spindle checkpoint–imposed arrest, wherein a cell exits mitosis without sister chromatid segregation or cytokinesis, ultimately yielding a mononucleated polyploid cell (Fig. 2). Mechanistically, although the spindle assembly checkpoint restrains APC/C‐driven progression, it does not fully prevent slow, progressive Cyclin B degradation during prolonged arrest. Once Cyclin B–CDK1 activity falls below the threshold required to sustain the mitotic state, cells reassemble a nuclear envelope and return to an interphase‐like condition with an unsegregated genome [38]. This process therefore represents a competition between mitotic‐exit kinetics and death‐in‐mitosis pathways, and slippage‐derived polyploidization occurs only if a cell can evade apoptosis long enough for CDK1 activity to drop beneath this critical threshold [38, 39].

**Fig. 2:**
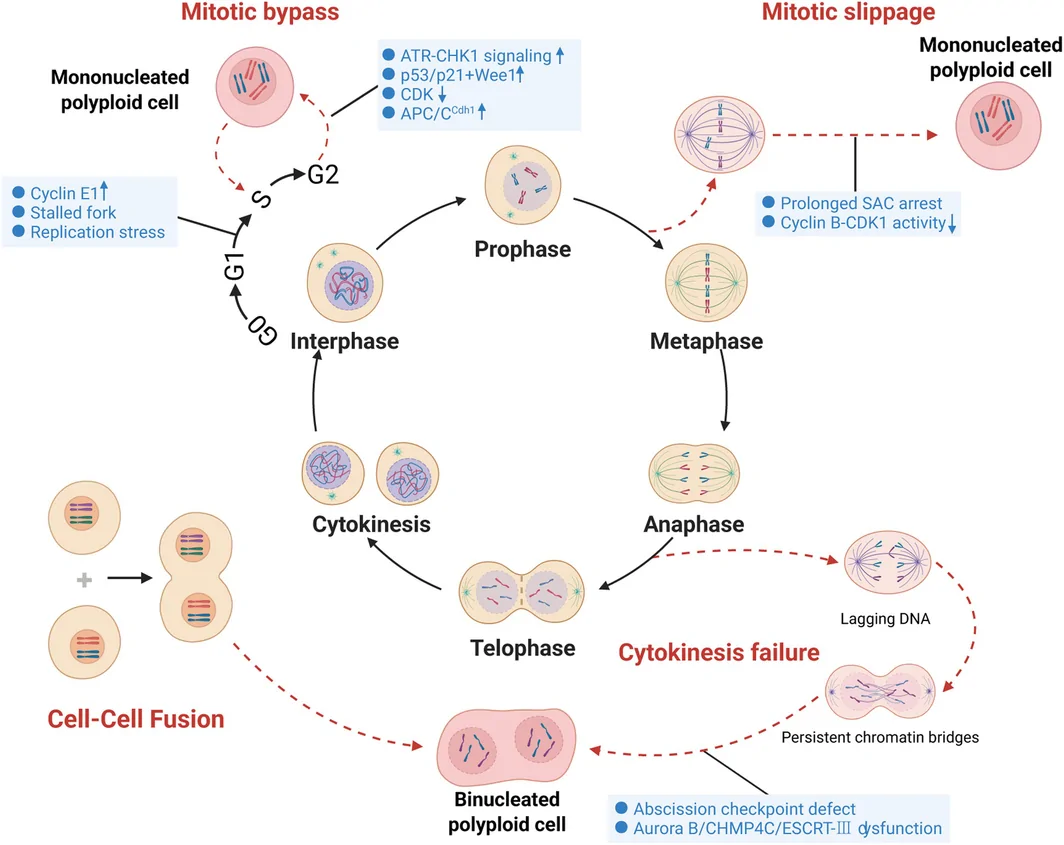
Schematic overview of canonical mechanisms leading to WGD. Schematic overview of four recurrent mechanisms that generate polyploid intermediates during WGD in cancer. (1) Mitotic bypass occurs when cells stall in G2 and re‐enter S phase without ever entering mitosis, driven by replication stress that suppresses mitotic kinase activity below the threshold for mitotic entry. (2) Mitotic slippage occurs when cells enter mitosis but exit without completing chromosome segregation or cytokinesis due to progressive loss of cyclin B‐CDK1 activity during prolonged spindle checkpoint arrest, yielding a mononucleated polyploid cell. (3) Cytokinesis failure occurs when chromosome segregation completes but cell separation fails due to persistent chromatin bridges or abscission checkpoint defects, generating a binucleated polyploid cell. (4) Cell–cell fusion merges two cells into a polyploid intermediate, although most fusion‐derived cells undergo catastrophic genomic instability and only a minority survive postfusion selection. Red dashed arrows denote aberrant routes to polyploidy that diverge from the normal cell cycle (solid black arrows). APC/C, anaphase‐promoting complex/cyclosome; ATR, ataxia–telangiectasia and Rad3‐related; CDK1, cyclin‐dependent kinase 1; CHK1, checkpoint kinase 1; CHMP4C, charged multivesicular body protein 4C; ESCRT‐III, endosomal sorting complex required for transport III; SAC, spindle assembly checkpoint; WGD, whole‐genome doubling.

#### 3.1.2 Cytokinesis failure and abscission checkpoint breakdown

Cytokinesis failure produces polyploid intermediates when chromosome segregation completes but separation of the two daughter cells fails, yielding a binucleated polyploid cell (Fig. 2). This failure is frequently triggered by chromatin bridges or lagging DNA trapped within the intercellular bridge [40, 41, 42]. These obstructions prevent the completion of abscission and can cause the cleavage furrow to regress, reuniting the two daughter cells into a single polyploid cell. Under normal conditions, the Aurora B‐dependent abscission checkpoint delays membrane scission by restraining the ESCRT‐III machinery until trapped chromatin is cleared, providing an opportunity to resolve the obstruction before abscission proceeds [40, 41]. In addition, loss of MSRB2, which normally maintains F‐actin polymerization within the intercellular canal, destabilizes the bridge and promotes furrow regression and binucleation specifically in cells harboring chromatin bridges [43].

#### 3.1.3 Replication stress‐driven mitotic bypass

Polyploidization can also arise through mitotic bypass, in which cells complete S phase but fail to enter mitosis and instead re‐enter a second round of DNA replication without chromosome segregation or cytokinesis (Fig. 2) [44]. Oncogene‐driven replication stress, particularly through CCNE1 deregulation, is a primary driver of this bypass [15]. CCNE1‐induced replication stress activates the ATR‐CHK1 pathway, forcing cells to arrest in G2 [15, 45]. In this arrested state, the p53‐p21 axis and Wee1 maintain sustained suppression of mitotic CDK activity, which in turn allows APC/C‐Cdh1 to reset the DNA replication machinery, preparing the cell for another round of DNA synthesis without an intervening division. Experimentally, this route is characterized by active DNA synthesis in polyploid cells lacking mitotic markers such as phospho‐histone H3 [15].

The route to polyploidization depends on the oncogenic context, and not all E‐cyclin family members act through the same mechanism. While CCNE1 deregulation primarily drives mitotic bypass through the pathway described above, it can alternatively trigger mitotic slippage in breast cancer models [46]. CCNE2 diverges further through a mechanistically distinct route. Unlike cyclin E1, cyclin E2 does not promote mitotic slippage but instead preferentially localizes to chromatin, where it associates with prereplication complex components MCM2 and MCM7. This chromatin‐bound CCNE2 promotes DNA rereplication, generating polyploid cells with subsequent negative feedback downregulation of the licensing factor Cdt1 [46].

#### 3.1.4 Cell fusion and posthybrid selection

Cell fusion provides an alternative route to WGD by merging two cells into a polyploid heterokaryon, a process facilitated by viral fusogens, inflammatory cytokines, or endogenous fusogenic proteins such as syncytins (Fig. 2) [47, 48]. This route is most relevant in virus‐associated cancers, including hepatitis‐driven hepatocellular carcinoma, EBV‐associated nasopharyngeal carcinoma, and HPV‐driven anogenital cancers [49]. In these settings, E6 and E7 disable p53‐ and pRb‐dependent polyploid checkpoints, respectively, while E5 promotes membrane fusion, collectively facilitating the generation of polyploid intermediates [50, 51, 52]. Importantly, fusion alone does not ensure a stable WGD lineage, because subsequent divisions of hybrid cells often involve severe spindle and segregation errors, and most hybrids undergo catastrophic genomic instability with only a minority surviving stringent posthybrid selection [53, 54].

### 3.2 Genetic determinants of WGD

#### 3.2.1 Cell division failure

WGD can arise when cells complete DNA replication but fail to complete division, doubling their genome without chromosome segregation or cytokinesis. Amplification of E‐cyclin family members exemplifies this process, with CCNE1 and CCNE2 driving WGD through distinct mechanisms (Table 1). CCNE1 amplification drives WGD through the replication stress‐induced mitotic bypass pathway, and notably, this pathway operates specifically in TP53‐wild‐type cells, as p53‐deficient cells instead enter catastrophic mitosis [15]. Consistent with this mechanism, CCNE1‐amplified tumors show a twofold higher likelihood of WGD compared to nonamplified tumors [12]. Unlike CCNE1, CCNE2 amplification drives WGD through a distinct rereplication mechanism, directly generating polyploid cells without mitotic bypass (Table 1) [46].

**Table 1:** Genetic determinants of whole‐genome doubling. This table summarizes representative genetic alterations that contribute to WGD through three functionally distinct roles. Checkpoint bypass alterations (TP53, RB1, CCND1, CDKN2A) disable the surveillance mechanisms that normally arrest or eliminate newly formed polyploid cells. Cell division failure alterations (CCNE1, CCNE2, BRAF, PIK3CA, FAT1) directly generate polyploid cells through mitotic bypass, DNA rereplication, or cytokinesis failure. The postpolyploid fitness alteration (PPP2R1A) helps genome‐doubled cells manage the structural consequences of extra centrosomes. For each gene, the key alteration, cancer context, reported WGD association, proposed mechanism, and supporting references are indicated.

Gene	Key alteration	Cancer type	WGD association	Role	References
TP53	Pathogenic/LoF mutations	Pan‐cancer	1.8× higher WGD overall in TP53‐mut vs. WT	Checkpoint bypass; removes polyploid production surveillance + tetraploid G1 arrest; structural mutants (R175H, V143A): complete arrest abolition; contact mutant (R273H): partial arrest retention	[12, 55, 56, 57, 58, 59]
RB1	Loss	Pan‐cancer	Altered in 5% of TP53‐WT WGD+ tumors	Checkpoint bypass; E2F derepression; G1/S arrest bypass	[12, 60]
CCND1	Focal amplification	Pan‐cancer	Altered in 7% of TP53‐WT WGD+ tumors	Checkpoint bypass; CDK4 hyperactivation; RB1 inactivation; G1 arrest override	[12, 60]
CDKN2A	Deletion	Pan‐cancer	Altered in 11% of TP53‐WT WGD+ tumors	Checkpoint bypass; CDK4/6 unleashing; RB1 inactivation; G1/S arrest bypass	[12, 60]
CCNE1	Amplification	Pan‐cancer	Altered in 1% of TP53‐WT WGD+ tumors	Mitotic bypass; replication stress‐induced G2 arrest; mitotic kinase decline; S phase re‐entry without mitosis; p53‐dependent	[12, 15]
CCNE2	Amplification/Overexpression	Breast cancer	16.4% of TCGA breast cancers amplified; 56% of amplified tumors genome‐doubled	DNA rereplication; chromatin‐associated rereplication without mitosis; p53‐independent	[46]
BRAF	V600E	Melanoma	WGD present in ~40% of melanomas	Cytokinesis failure; extra centriole production; RhoA suppression via RAC1; binucleate tetraploid formation	[61]
PIK3CA	H1047R (hotspot)	Breast cancer	H1047R mutations precede WGD, 13% of TP53‐WT WGD+ tumors	Cytokinesis failure; centrosome amplification via AKT; enhanced polyploid cell survival (60% vs 20% re‐entry)	[62, 63]
FAT1	Loss/Deletion	NSCLC	Positively selected before WGD, 5% of TP53‐WT WGD+ tumors	Dual mechanism; HR deficiency‐mediated replication stress + YAP1‐mediated cytokinesis failure; YAP1 alone sufficient; p53‐independent	[64]
PPP2R1A	P179R (hotspot)	Uterine carcinosarcoma	28% of uterine carcinosarcoma (WGD in 90% of cases)	Postpolyploid fitness; centrosome clustering; pseudo‐bipolar spindle formation; multipolar mitosis avoidance	[16, 24, 65, 66]

Cell division failure can also occur at a later stage of mitosis, when cells successfully segregate their chromosomes but fail to physically separate into two daughter cells, producing a binucleate tetraploid cell. Experimental studies have identified specific oncogenic mutations that cause this type of cell division failure. In melanoma, BRAF V600E disrupts the final step of cell division in two ways (Table 1). It induces supernumerary centriole formation and impairs the cytokinetic machinery required for physical separation by suppressing RhoA through RAC1 activation [61]. Studies in breast cancer have also linked PIK3CA H1047R to this process [62]. This mutation promotes centrosome amplification through AKT activation, disrupting the ROCK‐CDK2/cyclin E axis that normally limits centrosome duplication to once per cell cycle [62, 63]. After induced cytokinesis failure, H1047R‐mutant cells re‐entered division at three times the rate of wild‐type cells (60% vs 20%), indicating that this mutation not only generates polyploid cells but also enables their survival [62, 63]. Consistent with these findings, timing analysis of TCGA breast cancers showed that most H1047R mutations preceded WGD.

#### 3.2.2 Checkpoint bypass

Checkpoint bypass represents a fundamentally different route to WGD. Rather than causing cell division failure directly, these alterations disable the surveillance mechanisms that would normally detect and eliminate polyploid cells, allowing them to survive and re‐enter the cell cycle. TP53 is the most well‐established genetic determinant of this process. Wild‐type p53 normally prevents WGD at two levels. It arrests cells that acquire extra centrosomes or experience prolonged mitotic stress, limiting the production of polyploid intermediates [55, 56]. It also eliminates newly formed tetraploid cells through permanent arrest or apoptosis, preventing them from proliferating [1, 57, 58]. Loss of TP53 removes both barriers simultaneously, allowing polyploid cells to be generated more frequently and to survive and proliferate once formed (Table 1). This explains why TP53‐mutant tumors show an approximately 1.8‐fold higher likelihood of WGD than wild‐type tumors [12]. The impact of TP53 loss on WGD varies depending on the specific mutation. Functional studies have shown that structural mutants such as p53‐R175H and p53‐V143A act as dominant negatives that completely abolish tetraploid G1 arrest [59]. In contrast, p53‐R273H retains partial ability to maintain tetraploid arrest, so polyploid cells are not fully released from this checkpoint [59]. This difference may explain why tumors harboring R175H, such as certain high‐grade serous ovarian cancers, can maintain high‐ploidy states with relatively little subsequent LOH [67].

However, TP53 is not the only route. Approximately half of all WGD events occur in tumors with intact TP53 [12], pointing to additional mechanisms. In these tumors, WGD tolerance is achieved through defects in E2F‐mediated G1 arrest. For instance, RB1 loss, CDKN2A deletion, and focal CCND1 amplification are each independently associated with WGD, and 31.8% of TP53‐wild‐type WGD‐positive tumors harbor at least one such defect [12]. RB1 loss allows E2F‐driven gene expression to proceed unchecked, pushing cells past the G1/S boundary. CDKN2A deletion unleashes CDK4/6 activity, which in turn inactivates RB1 and produces the same unrestrained cell‐cycle entry. CCND1 amplification similarly overwhelms the G1/S checkpoint by hyperactivating CDK4, again disabling RB1 [12, 60]. In each case, newly formed polyploid cells are no longer arrested at G1/S and can re‐enter the cell cycle. Experimental studies confirmed this by showing that cyclin D overexpression can directly prevent newly formed tetraploid cells from arresting at G1 [60].

#### 3.2.3 Postpolyploid fitness

Even after escaping checkpoint arrest, polyploid cells face ongoing challenges from extra centrosomes and protein imbalance. Certain genetic alterations help them survive these stresses. The clearest example involves centrosome management. Functional studies showed that the recurrent PPP2R1A hotspot mutation p.P179R enhances centrosome clustering when centrosome number is increased, allowing genome‐doubled cells to build pseudo‐bipolar spindles and avoid lethal multipolar mitoses (Table 1) [65]. Consistent with these findings, pan‐cancer analyses identified PPP2R1A mutations as enriched in WGD‐positive tumors [16, 24].

## 4 Clinical landscape of WGD across cancers

### 4.1 Prevalence of WGD across cancer types

WGD occurs across a wide range of human cancers, but its frequency varies substantially by cancer type [2, 12, 68]. In a large‐scale sequencing study, the overall prevalence of WGD in primary tumors is approximately 30–40%, but individual cancer types range from 81% in esophageal cancer to 12% in glioblastoma multiforme (Fig. 3A) [69]. This variation reflects the distinct checkpoint and cell division defects permissive to WGD across different cancer types. TP53 loss is a common permissive event, but the overall WGD frequency in a given cancer type also depends on the presence of additional checkpoint defects and alternative routes to genome doubling.

**Fig. 3:**
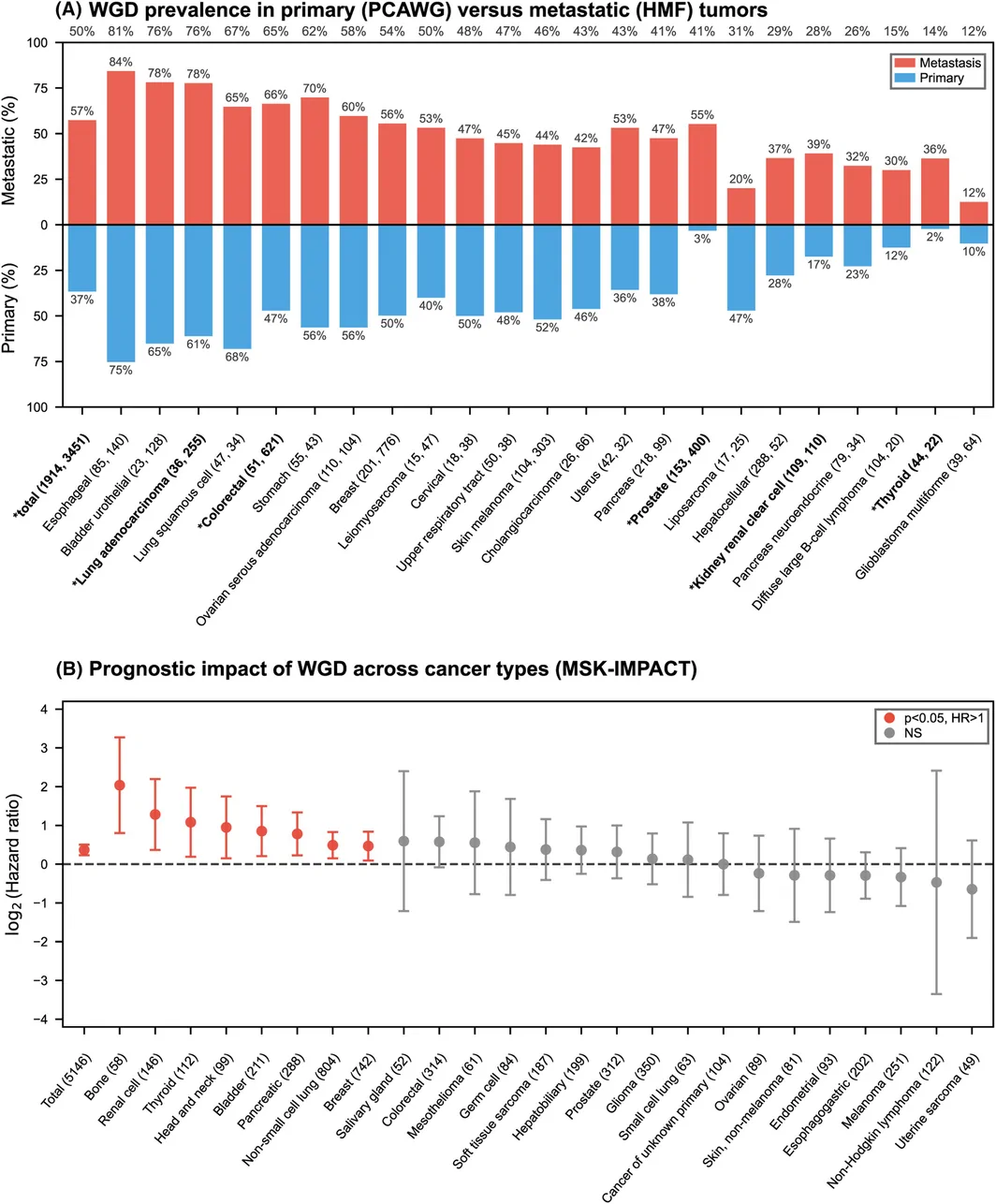
Clinical landscape of WGD across cancers. (A) Comparison of cancer‐type‐specific WGD prevalence between primary tumors from PCAWG and metastatic tumors from HMF [69]. Cancer types are ordered by pooled WGD prevalence across the compared cohorts. Parenthetical labels indicate the numbers of primary and metastatic samples. Numbers above the bars indicate the pooled percentage of WGD prevalence. Asterisks and bold labels indicate cancer types with a significant difference in WGD prevalence between the primary and metastatic cohorts. (B) Tumor‐type‐specific association of WGD with overall survival in the MSK‐IMPACT cohort from Bielski et al. [12], shown as hazard ratios (HRs) with 95% confidence intervals from univariate Cox models. Red points indicate cancer types with *P* < 0.05. HMF, Hartwig Medical Foundation; HR, hazard ratio; NS, not significant; PCAWG, Pan‐Cancer Analysis of Whole Genomes; WGD, whole‐genome doubling.

Esophageal carcinoma harbors one of the highest WGD frequencies at approximately 80%, paralleled by TP53 mutation rates of approximately 85% in this cancer type [70]. The strong association between TP53 loss and WGD is further supported by data from esophagogastric cancers, where approximately 80% of TP53‐mutant tumors harbor WGD [12]. Longitudinal analysis of Barrett's esophagus progression demonstrated that bi‐allelic TP53 loss precedes WGD, with TP53‐altered progressors showing a significantly higher WGD rate (35%) than nonprogressors (2.5%) [68]. Furthermore, WGD in esophageal carcinoma frequently co‐occurs with high‐level CCND1 amplification, suggesting that genome doubling facilitates the selection of oncogenic amplicons such as 11q13 during tumor progression [12, 70].

Bladder urothelial carcinoma also exhibits high WGD prevalence at 76.2% (Fig. 3A). Even at the nonmuscle‐invasive stage, WGD is observed in 15% of tumors, already accompanied by genomic instability, frequent 17p LOH affecting TP53, and recurrent alterations in cell‐cycle regulators including RB1, CCNE1, E2F3, and MYBL2 [71]. Notably, WGD‐positive nonmuscle‐invasive bladder cancer progresses to muscle‐invasive disease significantly faster than diploid tumors, indicating that WGD marks a more aggressive disease trajectory from an early stage [71]. As the disease progresses, this checkpoint‐permissive background becomes near‐universal, with the TP53/cell‐cycle pathway inactivated in 89% of muscle‐invasive cases [72].

Lung adenocarcinoma (75.6%) and lung squamous cell carcinoma (66.7%) similarly show high WGD prevalence (Fig. 3A). In the TRACERx lung adenocarcinoma cohort, truncal TP53 mutations significantly increase the likelihood of subsequent subclonal WGD and are associated with shorter disease‐free survival [73]. Beyond TP53, FAT1 alterations have been identified as another early driver that is positively selected before genome doubling, promoting WGD through replication stress and YAP1‐linked chromosomal instability [64]. Following these early events, WGD‐positive tumors diverge by subtype, with LUAD high‐ploidy clusters enriched for TP53 mutations and LUSC characterized by frequent CDKN2A/RB1 pathway disruption and focal amplifications involving SOX2, FGFR1, and CCND1 [74, 75].

In contrast to the cancer types described above, several other cancers show notably low WGD prevalence (Fig. 3A). This pattern can be explained by two factors. First, as discussed above, a low TP53 mutation frequency limits the background in which polyploid intermediates can survive and undergo clonal expansion. Second, alternative drivers such as focal amplifications, complex rearrangements, and strong oncogenic point mutations can already provide enough genomic complexity for tumor formation on their own. In such cases, the additional selective advantage of WGD is reduced [2, 21, 76]. GBM offers a clear example. Its TP53 mutation frequency is below 30% [77], and it also harbors many strong alternative drivers, including receptor tyrosine kinase amplifications and cell‐cycle pathway alterations. Both features are consistent with the low WGD prevalence observed in GBM [78].

Taken together, while TP53 inactivation provides a common permissive foundation for WGD across these cancer types, the specific co‐occurring alterations and evolutionary trajectories that drive high WGD frequency are cancer‐type‐specific.

### 4.2 WGD in primary versus metastatic tumors

A whole‐genome comparison of primary versus metastatic tumors demonstrated that WGD is more prevalent in metastatic tumors than in primary tumors for several cancers [69]. Among these, prostate cancer showed the most striking increase, from 3.3% in primary tumors to 55.2% in metastatic tumors. Whether WGD directly promotes metastatic dissemination remains unclear, but this enrichment is at least consistent with the selective progression of WGD‐positive primary tumors into metastatic disease, resulting in their over‐representation in metastatic cohorts [69]. A paired phylogenetic study of ten patients with lethal prostate cancer showed that, although primary tumors contained multiple coexisting clonal lineages, distant metastases consistently originated from a single lineage [79]. In one deeply characterized case, this metastasis‐founding lineage carried a focal loss of heterozygosity at PPP2R5A followed by WGD, whereas the remaining WGD‐negative lineages stayed confined to the prostate despite harboring other driver alterations [79]. This suggests that WGD arose not as a clonal event but within a specific subclone that went on to seed metastasis. However, the study was small, and the key mechanistic insight derives largely from a single patient. Thus, this finding should be regarded as a proof of principle rather than a universal rule for prostate cancer.

In lung adenocarcinoma, WGD is already common in primary tumors (61.1%) and increases further in metastatic disease (77.6%) (Fig. 3A). The key question in this cancer type is therefore whether genome doubling precedes metastatic seeding. The TRACERx study addressed this directly. Across 126 NSCLC tumors with paired primary and metastatic samples, clonal WGD was detected in 79 primary tumors, and in 64 of those 79 cases (81.0%), metastatic divergence occurred after the clonal WGD event [80]. These data indicate that in the large majority of WGD‐positive NSCLCs, genome doubling has already become a clonal event within the primary tumor by the time the metastatic lineage emerges.

In triple‐negative breast cancer, single‐cell DNA sequencing revealed that large‐scale copy‐number changes, including those associated with WGD, are established early in tumor development through punctuated bursts of genomic evolution rather than gradual accumulation [81]. These preconfigured clones subsequently seed lymph nodes and distant metastases with minimal additional genomic change, suggesting that the metastatic potential is embedded in the early genomic architecture of WGD‐positive clones [82]. A mechanistic basis for this association has been provided by experimental studies demonstrating that the CIN sustained in WGD‐positive cells generates micronuclei whose rupture activates the cGAS–STING cytosolic DNA‐sensing pathway, driving noncanonical NF‐κB signaling and EMT that promote cellular invasion and metastatic dissemination [13]. Although this mechanism was demonstrated using multiple cancer models, including breast and lung cancer cell lines, it establishes a general principle linking WGD‐driven CIN to metastatic competence.

Taken together, these findings converge on a model in which WGD establishes a genomic state that is permissive for metastasis. The specific relationship between WGD and metastatic progression varies by cancer type, ranging from subclonal acquisition in a metastasis‐founding lineage (e.g., prostate cancer) to early clonal fixation that precedes metastatic divergence (e.g., lung cancer) to preconfigured genomic architecture with a defined pro‐metastatic signaling mechanism (e.g., breast cancer).

### 4.3 Clinical significance of WGD as a prognostic biomarker

From a clinical perspective, WGD is increasingly recognized as a critical prognostic factor tightly linked to adverse survival outcomes. In the MSK‐IMPACT cohort, WGD was generally associated with poorer overall survival across multiple tumor types, with statistically significant associations observed in nonsmall‐cell lung, breast, bladder, pancreatic, prostate, thyroid, and bone cancers (Fig. 3B) [12, 83]. As discussed in Sections 1 and 2, WGD typically arises in a checkpoint‐permissive background and subsequently drives CIN and progressive accumulation of SCNAs. WGD therefore serves as a marker of a broader aggressive genomic trajectory in which weakened checkpoints permit genome doubling, and the resulting chromosomal instability generates the genomic diversity that underlies tumor progression.

In bladder cancer, WGD‐positive nonmuscle‐invasive tumors harbor high levels of genomic instability and widespread cell‐cycle checkpoint disruption, accompanied by an immune microenvironment enriched for T‐cell exhaustion markers [71]. The coexistence of unchecked genomic evolution and impaired immune surveillance likely accounts for the particularly aggressive behavior of WGD‐positive tumors in this cancer type.

In pancreatic adenocarcinoma, WGD is followed by broad copy‐number changes including large‐scale gains and losses that generate extensive genomic diversity [84]. In addition, allelic imbalance following WGD can increase mutant KRAS copy‐number dosage from early disease stages, and elevated KRAS dosage has been linked to more aggressive phenotypes and metastatic progression [85]. WGD in pancreatic cancer therefore marks a genomic state that both expands karyotypic diversity and amplifies oncogenic signaling, together contributing to its poor clinical outcome.

In breast cancer, the prognostic value of WGD is particularly evident within the ER+/HER2‐ subtype. This subtype generally exhibits low levels of genomic instability, but WGD‐positive tumors within this group display ongoing CIN and progressive accumulation of SCNAs that are more typical of genomically unstable cancer types [60]. WGD therefore identifies a high‐risk subset within an otherwise favorable molecular context, providing prognostic information that conventional classification alone does not capture.

Across these cancer types, the poor survival associated with WGD consistently coincides with underlying chromosomal instability, oncogene dosage amplification, and checkpoint disruption driven by genome doubling. However, the same mechanisms that allow tumors to tolerate the polyploid state also expose them to unique biological vulnerabilities, shifting the clinical focus from simply predicting a poor prognosis to actively exploiting these vulnerabilities for targeted therapeutic intervention.

## 5 Therapeutic vulnerabilities of WGD

### 5.1 Cell‐intrinsic vulnerabilities of WGD‐positive tumors

#### 5.1.1 Replication stress‐driven vulnerability

WGD does not itself constitute a direct therapeutic target, but the cellular stresses it imposes create dependencies that can be therapeutically exploited. The major vulnerability axes and their representative therapeutic strategies are summarized in Table 2. These dependencies are not strictly specific to WGD but are enriched in WGD‐positive tumors because genome doubling is a major source of the CIN, centrosome amplification, and aneuploidy that underlie them.

**Table 2:** Biological liabilities enriched in WGD/CIN‐high tumors and representative actionable nodes and strategy examples. This table summarizes treatment ideas by grouping WGD‐positive tumors according to the shared pressures that often increase with CIN and aneuploidy, rather than treating WGD as a single drug target. For each pressure type—replication stress/checkpoint dependence; mitotic accuracy and chromosome segregation control; centrosome‐clustering dependence; proteostasis/autophagy burden; and cytosolic DNA signaling and immune context—we list the target, representative agent(s), therapeutic rationale, and key references.

Vulnerability/immune state	Target	Representative agent(s)	Therapeutic rationale	References
Replication stress/checkpoint reliance	ATR, CHK1, WEE1	Ceralasertib, prexasertib, adavosertib	WGD/CIN‐high tumors experience elevated replication burden and checkpoint dependence; checkpoint inhibition may preferentially expose replication‐associated fragility	[23, 86, 87, 88]
Mitotic load/chromosome‐alignment dependency	KIF18A	Sovilnesib	CIN/aneuploidy‐high tumors become more dependent on chromosome‐alignment fidelity; KIF18A inhibition can convert alignment stress into mitotic catastrophe	[89, 90, 91]
Centrosome amplification/clustering dependency	KIFC1	CW069	Extra centrosomes create dependence on pseudo‐bipolar spindle maintenance; disrupting clustering may expose a lethal multipolar‐division phenotype	[11, 92, 93, 94, 95, 96]
Proteotoxic stress/proteostasis burden	Proteasome	Bortezomib	Aneuploid/WGD‐derived tumors accumulate proteome imbalance and may be more vulnerable to additional proteotoxic stress	[97]
Rewired STING‐intact immune state	IL‐6R/inflammatory rewiring axis	Tocilizumab	In selected CIN‐high contexts, chronic cGAS–STING output is rewired toward tumor‐promoting inflammatory signaling rather than productive interferon signaling	[30, 31]
Attenuated sensing/STING‐low immune state	DNMTi‐based immune priming	Decitabine	In ovarian/WGD‐relevant STING‐low contexts, epigenetic immune priming may help restore or bypass attenuated sensing states	[14, 32, 98, 99, 100, 101]

WGD increases the total amount of DNA that must be replicated each cell cycle, and when combined with oncogene‐driven replication stress, this elevated burden makes WGD‐positive cells more prone to replication fork collapse and DNA damage [15]. Consistent with this, isogenic polyploid models show that WGD‐positive cells are preferentially sensitive to depletion of the replication stress response genes RRM1 and RAD51, and to the replication stress‐inducing agents– hydroxyurea and gemcitabine [16]. Independent work confirmed that newly formed polyploid cells experience replication‐associated DNA damage during their first post‐WGD S phase, indicating that this vulnerability arises immediately after genome doubling [23]. In the many WGD‐positive tumors that also lack functional p53, cells cannot halt the cell cycle when replication errors occur and become critically dependent on the ATR‐CHK1‐WEE1 axis to manage ongoing replication damage [86, 87, 88]. Disrupting this checkpoint with ATR, CHK1, or WEE1 inhibitors could therefore force such cells into mitosis with unresolved DNA damage, a condition that is selectively lethal in cells already burdened by elevated replication stress. However, because these agents target a general DNA damage response pathway, patient selection will likely need to integrate WGD status with the degree of replication stress and the integrity of remaining DNA repair pathways.

#### 5.1.2 Mitotic fidelity‐associated vulnerability

Beyond replication, WGD increases the physical burden of chromosome segregation during mitosis. The doubled chromosome complement raises the frequency of kinetochore‐microtubule attachment errors, particularly merotelic attachments, which promote chromosome mis‐segregation [11, 102]. Because CIN/aneuploidy‐high tumors already operate near the upper limit of tolerable mis‐segregation, they have less margin to absorb additional mitotic errors than chromosomally stable cells [89, 102]. This makes them more dependent on the mechanisms that maintain chromosome alignment and more vulnerable when those mechanisms are disrupted. Accordingly, aneuploid cancer cells are selectively sensitive to mitotic checkpoint inhibition [89]. Among distinct effectors, the mitotic kinesin KIF18A has emerged as a key dependency, as chromosomally unstable tumor cells specifically require KIF18A for proper chromosome alignment and proliferation [90, 91]. Small‐molecule KIF18A inhibitors (e.g., sovilnesib/AMG‐650 and VLS‐1488) exploit this selective dependency and are currently in early‐phase clinical trials [91, 103, 104, 105]. While the primary selection biomarker for these inhibitors is CIN or aneuploidy status, WGD can serve as a clinically accessible surrogate for identifying tumors with the high levels of chromosomal instability that predict sensitivity.

#### 5.1.3 Centrosome amplification‐driven vulnerability

WGD frequently generates supernumerary centrosomes, which expose cells to multipolar spindle formation and catastrophic chromosome mis‐segregation. As a result, many polyploid cells rely on centrosome clustering to gather extra centrosomes and perform pseudo‐bipolar division [11, 92]. Because centrosome clustering is mechanistically linked to the structural output of WGD rather than to general CIN, it serves as a WGD‐specific hallmark therapeutic vulnerability. A genome‐wide RNAi screen confirmed this by showing that knockdown of key clustering factors selectively kills cells with extra centrosomes [92, 93]. Although several motor and cortical linker mechanisms contribute to clustering, most are also essential for normal mitosis, leaving a narrow window for selective targeting. By contrast, KIFC1 is a kinesin‐14 motor that crosslinks antiparallel microtubules emanating from different centrosomes and pulls them inward toward their minus ends, coalescing extra centrosomes into two functional spindle poles [93]. As a result, KIFC1 (also known as HSET) is selectively required only in cells that have undergone WGD, making it a genuinely actionable target in this category. This mechanism is distinct from other mitotic motor dependencies such as KIF11‐mediated spindle bipolarization or CENPE‐mediated chromosome congression and kinetochore‐microtubule attachment [106, 107]. KIFC1 dependency is instead mechanistically coupled to the centrosome amplification caused by WGD and therefore represents a more WGD‐specific axis among mitotic motor targets. Pharmacological inhibition of KIFC1 with the allosteric probe CW069 or the small‐molecule inhibitor AZ82 preferentially induces multipolar mitosis in cells with supernumerary centrosomes [94, 95, 96]. These inhibitors remain at the preclinical stage, but WGD status could help identify tumors most likely to depend on centrosome clustering for survival.

#### 5.1.4 Aneuploidy‐driven proteostatic vulnerability

Finally, WGD and the ongoing aneuploidy it generates impose a chronic burden on protein homeostasis. Stoichiometric imbalance across multi‐protein complexes caused by unbalanced chromosome gains and losses increases the load on protein folding and clearance pathways, leading to proteotoxic stress [26, 27, 108]. Functional genetic screens in isogenic WGD models directly demonstrated that WGD‐positive cells are more dependent on proteasome function than their diploid counterparts [16], consistent with earlier work showing that aneuploid cells are selectively sensitive to compounds that exacerbate protein‐folding stress [27]. This dependency extends to the clinically approved proteasome inhibitor bortezomib, where aneuploidy levels predict drug sensitivity both in cancer cell lines and in patients with multiple myeloma [97].

Taken together, these four vulnerability axes (Fig. 4A) are not mutually exclusive and may co‐exist within the same tumor, suggesting that rational combination strategies targeting multiple WGD‐associated dependencies simultaneously could achieve greater therapeutic efficacy than single‐agent approaches. Effective clinical translation will require integrating WGD status with complementary biomarkers, including CIN metrics, TP53/DDR pathway status, and measures of replication stress, to match each tumor to its most actionable vulnerability.

**Fig. 4:**
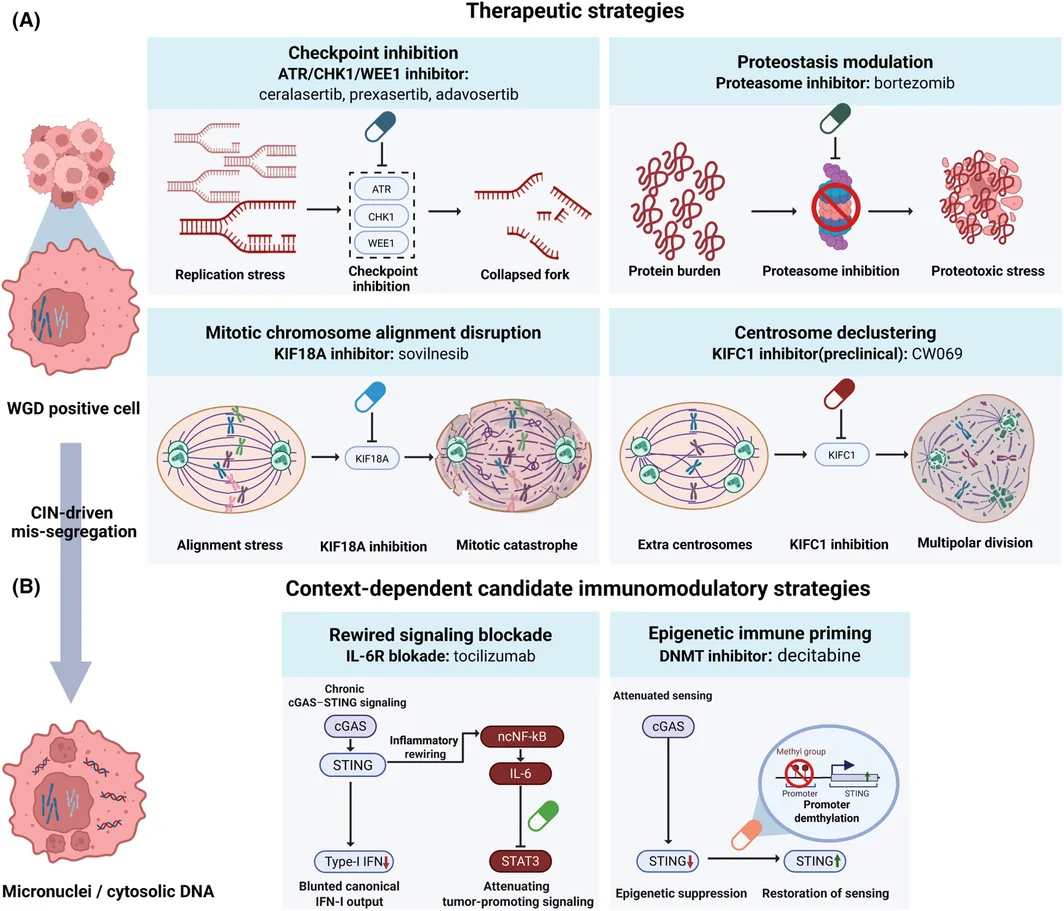
Therapeutic vulnerabilities in WGD tumors. (A) Cell‐intrinsic vulnerabilities include replication stress/checkpoint dependence, mitotic chromosome‐alignment and centrosome‐clustering dependencies, and proteostasis burden, with representative targets and agents shown. (B) CIN‐driven micronuclei/cytosolic DNA can be associated with distinct immune states, including rewired cGAS–STING‐associated inflammatory signaling and attenuated sensing states, with illustrative candidate immunomodulatory strategies shown. CIN, chromosomal instability; DNMTi, DNA methyltransferase inhibitor; IFN, interferon; IL‐6R, interleukin‐6 receptor; ncNF‐κB, noncanonical NF‐κB; WGD, whole‐genome doubling.

### 5.2 Immune‐modulatory vulnerabilities of WGD‐positive tumors

#### 5.2.1 Targeting rewired cGAS–STING signaling

Whole‐genome doubling reshapes the tumor–immune interface both through cell‐intrinsic alterations and through the persistent CIN it generates. Because much of the immune‐modulatory literature is framed in terms of CIN rather than WGD specifically, the two states warrant explicit distinction at the outset. CIN refers to ongoing chromosome mis‐segregation or to the cumulative copy‐number burden that can arise after WGD but can also arise through WGD‐independent mechanisms. In contrast, WGD denotes a discrete genome‐state transition that doubles the chromosome complement and alters cell size, centrosome number, and replication load. KIFC1 and similar vulnerabilities depend on cellular states produced by WGD itself. Even when tumors show similar CIN burden, their reliance on these vulnerabilities can differ depending on whether the CIN originated from a WGD event. Consistent with this, analysis of the MSK‐IMPACT cohort showed that WGD retained independent prognostic value even after adjustment for clinical and genomic covariates, indicating that WGD reports outcome information not captured by CIN burden alone [12]. Therefore, the CIN‐focused immune studies cited below provide mechanistic support for pathways likely to be sustained in WGD‐driven CIN, rather than evidence that WGD alone is sufficient as a clinical selection biomarker.

Beyond cell‐intrinsic dependencies, the CIN driven by WGD also shapes the tumor–immune interface in ways that create distinct therapeutic opportunities. Because WGD is a major source of persistent CIN, the immune consequences of CIN are directly relevant to WGD‐positive tumors. In some CIN‐high tumors, cGAS–STING signaling remains active but its output is redirected away from productive antitumor immunity toward inflammatory programs that support tumor cell survival. In triple‐negative breast cancer, this takes the form of IL‐6‐STAT3 activation through NF‐κB, and blocking IL‐6 signaling with the receptor antibody tocilizumab selectively impaired the growth of CIN‐positive but not CIN‐negative tumors both *in vitro* and *in vivo* [31]. Independently, in metastatic melanoma, breast, and colorectal cancer models, chronic CIN was shown to desensitize type I interferon responses downstream of STING, while NF‐κB‐driven inflammatory programs became dominant [30]. In these models, inhibiting STING at its source suppressed CIN‐driven metastatic progression, representing a distinct approach from the downstream effector blockade exemplified by tocilizumab [30]. Although these studies did not directly assess WGD status, the persistent CIN that characterizes WGD‐positive tumors is precisely the condition that sustains this rewired signaling state, making WGD‐positive tumors plausible candidates for either upstream STING antagonism or downstream inflammatory pathway blockade.

#### 5.2.2 Targeting attenuated immune sensing in WGD tumors

In contrast to the rewired state, other CIN‐high tumors lose cGAS–STING sensing capacity itself, meaning the tumor fails to detect its own chromosomal damage. In this context, the most direct WGD‐specific evidence comes from high‐grade serous ovarian cancer. Single‐cell WGS analysis showed that WGD status can be assessed at the level of individual cells, allowing tumor samples to be classified as WGD‐high or WGD‐low based on the proportion of cells that have undergone WGD. Under this classification, WGD‐high HGSOC tumors have increased chromosomal mis‐segregation and a 3.3‐fold higher frequency of micronuclei, yet display lower STING1 expression, reduced type I interferon signaling, and a more immunosuppressive microenvironment than WGD‐low tumors [14].

A plausible mechanism for this loss is epigenetic silencing. Pan‐cancer analyses have shown that cGAS and STING promoters are frequently silenced through promoter methylation across multiple tumor types [32, 98], providing a mechanistic framework that may explain the STING1 loss observed in WGD‐high ovarian tumors. In ovarian cancer specifically, treatment with the DNA‐demethylating agent 5‐aza‐2′‐deoxycytidine restored STING/cGAS expression and downstream interferon responses [99, 100, 101], suggesting that epigenetic immune priming could restore upstream sensing in STING‐low WGD‐high tumors [14, 99]. Notably, WGD timing may also influence which immune state a tumor adopts. Tumors with earlier WGD show greater depletion of MHC class II expression than those with late WGD [19], suggesting that prolonged post‐WGD evolution provides more opportunity for immune‐evasive remodeling, including epigenetic silencing of innate sensing pathways.

The distinction between these two immune states has direct therapeutic implications (Fig. 4B). In tumors with rewired but active cGAS–STING signaling, the therapeutic goal is to block aberrant inflammatory output, either at the source through STING antagonism or at the effector level through IL‐6R blockade. In tumors with attenuated sensing, the goal is fundamentally different—to restore upstream cGAS–STING function through epigenetic immune priming. Whether WGD status, potentially combined with STING1 expression, promoter methylation, or WGD timing, can serve as a stratification biomarker to distinguish these two contexts remains an important open question.

## 6 Conclusions and future perspectives

In this review, we have examined WGD as an integrated biological phenomenon, connecting the mechanisms that generate and sustain genome doubling to its clinical consequences and emerging therapeutic opportunities. The genome‐doubled state amplifies cellular stresses beyond those found in diploid tumors, and these stresses have two opposing consequences. They drive the aggressive phenotypes that account for the poor prognosis of WGD‐positive cancers, including the persistent CIN that fuels tumor evolution. At the same time, these stresses force WGD‐positive cells into a heightened dependence on specific survival mechanisms. Poor prognosis and therapeutic vulnerability in WGD‐positive tumors therefore arise from the same underlying biology, and identifying a tumor as WGD‐positive is not simply a prognostic statement but an entry point for targeting the specific dependencies that the genome‐doubled state creates.

However, using WGD as a guide for treatment decisions is not yet straightforward. The same WGD event can lead to different vulnerabilities in different tumors, depending on which checkpoints are intact, how much chromosomal instability is present, and the state of immune sensing pathways [12, 14]. The distinction between rewired and attenuated cGAS–STING states is particularly consequential, as these two contexts require fundamentally opposing therapeutic strategies, with downstream inflammatory blockade appropriate for the former and epigenetic immune priming for the latter. Adding to this complexity, single‐cell studies have revealed that WGD is not always a single tumor‐wide event but can occur at different times and in different subclones within the same tumor [14, 81], with earlier WGD associated with more extensive immune evasion [19] and with WGD‐positive subclones disproportionately contributing to metastatic lineages in certain cancer types [79, 80]. Currently, no biomarker captures these variables together, leaving clinicians without a systematic way to determine which vulnerability a given WGD‐positive tumor is most likely to harbor.

To bridge this translational gap, the most immediate priority is developing integrated biomarker frameworks that combine WGD status with checkpoint genotype, CIN level, and immune context such as STING1 expression or promoter methylation status. Several agents targeting the cell‐intrinsic dependencies described in this review, including ATR/CHK1/WEE1 inhibitors and KIF18A inhibitors, are now in early‐phase clinical trials [86, 87, 88, 91, 103, 104, 105], but WGD or CIN status has not yet been used to prospectively select patients for these interventions. Incorporating these molecular features into future trial designs and demonstrating that they can identify likely responders in a prospective setting remains an essential and unmet goal.

WGD can already be inferred from the sequencing data routinely generated in clinical oncology, and the biological rationale linking genome doubling to specific therapeutic vulnerabilities is now supported by converging preclinical and translational evidence. Among the sequencing platforms available in clinical practice, no study has directly compared their WGD detection performance head‐to‐head. Based on their technical properties, however, WGS appears to provide the most stable WGD detection. This is because WGS offers uniform coverage across the entire genome and dense SNP information, enabling accurate allele‐specific copy‐number analysis [109]. By contrast, whole‐exome and targeted panel data have nonuniform coverage and capture only a small number of informative heterozygous SNPs, which can lead to ambiguous ploidy solutions and may underestimate WGD [110, 111]. Consistent with this, the PCAWG and HMF cohorts analyzed by WGS [69] report higher WGD frequencies than the MSK‐IMPACT advanced cancer cohort analyzed by targeted panel sequencing [12]. Across all platforms, ploidy estimation is also substantially influenced by tumor purity, a factor that should be appropriately accounted for in WGD calling. Despite these technical considerations, recent studies have shown that WGS can be applied to routine clinical diagnostics [112]. As WGS becomes more widely used in clinical practice, more accurate WGD detection will likely become increasingly feasible.

What remains to be established is whether integrating WGD status and its molecular context into clinical decision‐making can improve treatment outcomes. The framework outlined here, integrating WGD status with checkpoint genotype, CIN burden, immune sensing state, and WGD timing into a unified stratification model, provides a roadmap for realizing the potential of WGD as a precision medicine biomarker in cancer.

## Conflict of interest

The authors declare no conflict of interest.

## Author contributions

SJL: writing – review and editing, writing – original draft, visualization. JHL: writing – review and editing, visualization. JHB: writing – review and editing, writing – original draft, supervision, funding acquisition, conceptualization.
